# Dynamics of psychological distress: understanding the impact of intraindividual and interindividual factors in the Belgian population during the COVID-19 pandemic—a multilevel prospective cohort study

**DOI:** 10.3389/fpsyt.2026.1716253

**Published:** 2026-03-06

**Authors:** Camille Duveau, Pablo Nicaise, Pierre Smith, Katharina Seeber, Richard Bryant, Giovanni Corrao, Mireia Félez-Nóbrega, Josep Maria Haro, Irwin Hecker, Kerry Rodríguez McGreevy, Roberto Mediavilla, Maria Melchior, Ellenor Mittendorfer-Rutz, Matteo Monzio Compagnoni, Papoula Petri-Romão, Antje Riepenhausen, Jutta Stoffers-Winterling, Anke Witteveen, Marit Sijbrandij, Vincent Lorant

**Affiliations:** 1 Institute of Health and Society (IRSS), Université catholique de Louvain (UCLouvain), Brussels, Belgium; 2 School of Psychology, University of New South Wales, Sydney, NSW, Australia; 3 Unit of Biostatistics, Epidemiology and Public Health, Department of Statistics and Quantitative Methods, University of Milano-Bicocca, Milan, Italy; 4 National Centre for Healthcare Research and Pharmacoepidemiology, University of Milano-Bicocca, Milan, Italy; 5 Research and Development Unit, Parc Sanitari Sant Joan de Déu, Sant Boi de Llobregat, Spain; 6 Centre for Biomedical Research on Mental Health (CIBERSAM), Madrid, Spain; 7 Département d'Epidémiologie Sociale, Institut Pierre Louis d'Epidémiologie et de Santé Publique, Institut national de la santé et de la recherche médicale (INSERM), Sorbonne Université, Paris, France; 8 Department of Psychiatry, Universidad Autónoma de Madrid (UAM), Madrid, Spain; 9 Instituto de Investigación Biomédica del Hospital Universitario La Paz (IdiPAZ), Madrid, Spain; 10 Leibniz Institute for Resilience Research, Mainz, Germany; 11 Department of Clinical Neuroscience, Division of Insurance Medicine, Karolinska Institutet, Stockholm, Sweden; 12 Department of Psychiatry and Neurosciences CCM, Research Division of Mind and Brain, Charité, Berlin, Germany; 13 Universitätsmedizin Berlin & Berlin School of Mind and Brain, Faculty of Philosophy, Humboldt-Universität, Berlin, Germany; 14 Department of Clinical, Neuro- and Developmental Psychology, WHO Collaborating Centre for Research and Dissemination of Psychological Interventions, VU University, Amsterdam, Netherlands

**Keywords:** Belgium, COVID-19, loneliness, mental health, psychological distress

## Abstract

**Introduction:**

Longitudinal studies have identified an increase in psychological distress throughout the general population during the COVID-19 pandemic. Nevertheless, the determinants of the variation in psychological distress are unclear. This paper investigated factors that were likely to be associated with psychological distress variation: exposure to COVID-19 and psychosocial factors.

**Methods:**

Five waves of a prospective cohort survey were conducted with a convenience sample of the general population in Belgium between March 2020 and November 2021 (n=4,550). Psychological distress was measured using the GHQ-12. Two groups of exposures were investigated: self-reported exposure to COVID-19 and psychosocial factors (loneliness, social support, and social activities). We first partitioned the variance into an interindividual component (time-invariant) and an intraindividual component (time-variant). Linear mixed models were used for analysis.

**Results:**

Most of the variance in psychological distress was interindividual. For both sources of variance (interindividual and intraindividual), the change in psychological distress was mainly associated with psychosocial factors, rather than pandemic-related factors. Loneliness emerged as the factor most strongly associated both with interindividual and intraindividual differences in psychological distress.

**Discussion:**

Overall, these findings suggest that the variation in psychological distress between the waves was mostly influenced by social support, loneliness, and social activities, rather than by exposure to COVID-19. Mitigation policies aimed at controlling the pandemic should focus more on addressing specific individual psychosocial vulnerabilities.

## 1 Introduction

Numerous systematic reviews investigating the mental status of populations during the COVID-19 pandemic period revealed elevated self-reported levels of psychological distress compared to pre-pandemic levels (1, 2). Penninx and colleagues, however, found that high levels of self-reported mental health issues did not translate into increased rates of psychiatric disorders globally (3). From the first studies published, certain specific demographic groups have been identified as vulnerable to psychological distress in pandemic conditions, including women and young people (4–7), individuals from lower socioeconomic backgrounds (8), individuals with limited social support (7), and people experiencing high levels of loneliness (9, 10). Individual characteristics, such as sociodemographic and economic status, explained roughly 30% of the variation in psychological distress (11).

The rise in psychological distress was linked to both infection rates and restrictions on social contacts and mobility. Aknin et al. found that stricter COVID-19 policies were associated with higher psychological distress, controlling for individual and contextual variables (12). However, another study reported only modest effects of lockdowns on mental health in the general population (13). In the United States, Breslau et al. concluded that as many people experienced serious psychological distress in 30 days during the pandemic as in an entire pre-pandemic year (14). In Germany, Mata et al. noted an initial distress spike, followed by stabilization or slight decline (15). Several studies found psychological distress fluctuated with suppression policies (7, 10, 16), while some suggested resilience emerged weeks after lockdowns (17, 18). The effects of policy measures varied by context and individual factors, leaving uncertainty between psychosocial factors and political restrictions. Identifying the predictors of longitudinal variation is crucial for mitigating the mental health consequences of the pandemic.

Previous studies have focused on the pandemic’s direct effect, with little attention to intraindividual and interindividual variations in psychological distress over time. In this paper, *interindividual* variation refers to differences between individuals (due to, for example, stable sociodemographic characteristics, such as age, gender, and level of education). By contrast, *intraindividual* variation refers to differences within individuals over time (due to characteristics that are likely to change, such as social activities, loneliness, social support, and exposure to COVID-19). Intraindividual variation was affected by policy measures, unlike interindividual variation. Loneliness, defined as a dissonance between one’s desired and actual social connections, is related to anxiety, depression, and unhealthy behaviors, especially for the most vulnerable population (12, 19, 20). Loneliness was also a direct consequence of suppression policies (21). This study aims to assess how intra- and interindividual variations, influenced by psychosocial factors (e.g. loneliness, social support, social restrictions), explain psychological distress during the pandemic. Specifically, we examined how psychological distress was associated with (1) interindividual (time-invariant) and intraindividual (time-varying) variance, (2) with psychosocial factors (e.g. restrictions on social support, loneliness, social activities), and (3) with exposure to COVID-19.

Finally, this study could aid in identifying groups that are more vulnerable to the detrimental effects of loneliness. This study could also be valuable for better understanding the effects of the pandemic on mental health and for designing policies to prepare for future pandemics.

## 2 Materials and methods

### 2.1 Settings

The study was conducted in Belgium, one of the most severely impacted countries by COVID-19, with 830 deaths per million inhabitants between April and August 2020, compared to 456 in France, 574 in Italy, and 593 in the UK (4, 22, 23). Belgium implemented a lockdown on March 13, 2020, with major restrictions including a “social bubble” (limiting the number of people in a household could mix with), mandatory teleworking, closure of non-essential businesses and travel limits. Most restrictions were progressively lifted in May and June 2020 as infection rates decreased. However, a second wave in September 2020 led to the reinstatement of most restrictive measures in November, including a curfew, lasting until June 2021.

### 2.2 Design

To track mental health trends over time, the “*COVID-and-I*” longitudinal online survey was launched in March 2020, just two days after the first lockdown began. A convenience sampling method was employed. An online survey was promoted through social media, national newspapers, radio, and television advertisements. Respondents provided digital informed consent and provided their e-mail addresses for follow-up. The study consisted of five waves conducted between 2020 and 2021: March 2020, April 2020 (during the first wave of outbreaks and lockdown), June 2020 (after restrictions eased), November 2020 (during the second wave of outbreaks and restoration of restrictive measures), and November 2021 (five months after lockdown measures eased, in June 2021). Detailed methodology is available in prior publications (4, 22). The participant count for each wave is provided **Table 1**.

**Table 1:** Waves 1 to 5 of the COVID-and-I survey: number of participants in each wave.

Wave	Date	Number of participants
Wave 1	March 2020	N= 21,734
Wave 2	April 2020	N= 12,248
Wave 3	June 2020	N= 9,685
Wave 4	November 2020	N= 7,600
Wave 5	November 2021	N= 6,759

### 2.3 Participants

In March 2020 (Wave 1), 21,734 participants responded to the survey, with 14,094 providing their email addresses for follow-up. Subsequently, 56.3% of the initial respondents took part in April 2020 (Wave 2), followed by 44.6% in June 2020 (Wave 3), 35.0% in November 2020 (Wave 4), and 31.1% in November 2021 (Wave 5). After excluding responses with missing data on psychological distress, the final sample comprised 4,550 participants who completed all five study waves, representing 21% of the initial sample.

### 2.4 Measures

#### 2.4.1 Psychological distress

Our dependent variable, psychological distress, was assessed using the 12-item General Health Questionnaire (GHQ-12), which captures common mental disorders with scores ranging from 0 (no psychological distress) to 12 (severe psychological distress) (24). The scale displays good psychometric properties, with a Cronbach’s alpha of 0.90 (25). The GHQ was analyzed as a continuous score indicating the magnitude of distress. A score of 4 or higher indicates non-psychotic mental illness (24, 26).

The study also included socioeconomic factors that generally present little variation over time, such as age at baseline (in years), gender (men or women), and education level at baseline (classified as lower (e.g. primary school) or higher).

Conforming to our model, two groups of time-varying factors were included: exposure to COVID-19 and psychosocial factors. Exposure to COVID-19 was assessed using three yes/no questions: whether the participant was infected, lived with someone infected, or had a close relative/friend infected. Those answering “yes” to any were considered exposed (4).

Psychosocial factors included social support, loneliness, and number of social activities, measured across five waves. Social support was assessed using the 3-item Oslo Social Support Scale, which generates a score ranging from 3 (indicating poor social support) to 14 (indicating strong social support) (27). Loneliness was measured using the Short Loneliness Scale (LON), which yields a score ranging from 3 (indicating a low level of loneliness) to 12 (indicating a high level of loneliness) (28).

The number of social activities was assessed using an adapted version of the Social Participation Measure (SPM), ranging from 6 to 24, based on frequency of participation in six categories of social activity during the survey week (29). Those activities were rated on a scale ranging from 1 (never) to 4 (four or more times a week), and included 1) visiting relatives, friends, or acquaintances, 2) attending spectacles, conferences, lectures, concerts, or sports events, 3) practicing sport, theatre, music, and other hobbies, 4) going to a pub, bar, restaurant, club, party, or picnic, 5) performing social errands (shopping, going to the doctor, the lawyer, etc.), and 6) participating in activities at home, such as reading a book, watching a movie, or listening to music.

#### 2.4.2 Pandemic waves

Finally, the study used data from five waves (March 2020, April 2020, June 2020, November 2020, and November 2021) to track changes over time, reflecting the evolving pandemic and shifting public health measures, societal responses, and the overall impact of COVID-19 over time.

### 2.5 Statistical analysis

The primary analysis employed a complete-case approach, selecting participants who had completed all five waves, yielding a final sample of 4,550 participants. To address potential selection bias, a supplementary analysis included all participants who participated in at least one wave (n=22,750), and the results were compared with the complete-case analysis (see **Supplementary Table 1**).

Sample means and proportions were described, using the Chi-squared test for discrete variables (i.e. exposure to COVID-19) and the F-test for continuous variables (i.e. psychological distress, social support, loneliness, and social activities).

Acknowledging the strong correlation between an individual’s current and past mental health status, we used a linear mixed-effect model. We employed a Bayesian linear mixed-effects model to analyze the data, using the BGLIMM procedure in SAS (version 9.4). This approach was chosen because it provides more robust estimations when dealing with unbalanced data, and is well-suited for complex hierarchical models, in which it can easily accommodate a high number of random (intercept and slope) effects (30).

Continuous outcomes for the GHQ-12 score were used to model the association between the predictor variables and psychological distress. Random effects allowed for different intercepts and slopes (wave and loneliness level), creating a specific model for each participant.

Random intercepts (S0s) captured interpersonal differences for the whole period covered, whereas random slopes (S1s) captured intrapersonal differences between time points. The random slope for wave allowed individuals to differ in their changes across waves, thereby modelling heterogeneity in longitudinal trajectories. The random slope for loneliness captures heterogeneity in the effect of loneliness on mental health outcome between individuals, indicating that increase in loneliness is associated with larger changes in the outcome for some individuals compared to others. Thus, this random effect indexes individual differences in vulnerability to loneliness with steeper slopes reflecting greater sensitivity to loneliness and flatter slopes indicating relative resilience. This approach facilitated an examination of how various factors influenced both overall psychological distress levels (random intercepts) and the changes in psychological distress between the study waves (random slopes). Model 1 included the socioeconomic characteristics (gender, age, and education) as fixed effects, with wave variables as both fixed and random effects, plus a random intercept to account for individual variance. Model 2 added exposure to COVID-19 as a fixed effect. Model 3 incorporated psychosocial variables, including level of social support, loneliness, and social activities. Model 3B, presented in **Supplementary Table 2**, included interaction effects between loneliness and gender or age. Model 4 added a random slope for loneliness capturing how vulnerable different individuals are to loneliness.

To evaluate the explained variance in relation to added complexity, we compared model fits using the Deviance Information Criterion (DIC). DIC is similar to other information criteria such as the Akaike Information Criterion (AIC) and Bayesian Information Criterion (BIC): lower DIC values suggest a better-fitting model. DIC includes a penalty for model complexity to avoid overfitting. It accounts for the number of effective parameters in the model, balancing goodness of fit with model simplicity (30). Descriptive analyses and mixed-effects models were performed using an alpha level of 0.05. For the Bayesian mixed-effects models, the precision of posterior mean parameter estimates was indicated using 95% credible intervals (95% CIs). These 95% credible intervals are provided for all models. The 95% credible intervals directly quantify the probability that a parameter lies within a given range, conditional on the observed data and the specified prior.

To visually represent the transition in psychological distress status over the study waves, a Sankey graph was employed, using the GHQ-12 score cut-off point of 4. Furthermore, contrast tests were conducted to assess interactions between gender/age differences and the changes observed in psychological distress over the study waves.

## 3 Results

The sociodemographic characteristics of the participants at baseline and descriptive data of various factors are presented in **Table 2**. The sample consisted of 4,550 respondents to all five waves, with an average age of 50.5 years (SD = 14.1). Of that sample, 72.5% were women and 89.6% had a high level of education (secondary school or higher). At the outset of the study in March 2020, the mean psychological distress score was 3.97 out of 12 (SD = 3.39), just below the GHQ-12 threshold for psychological distress (≥4). Roughly 47% of respondents reported a GHQ score ≥4 in that wave, as depicted in the Sankey graph illustrated in **Supplementary Figure 1**. Subsequently, during the first lockdown period in April 2020 (Wave 2), the average psychological distress score increased to 4.08 (SD = 3.75). As shown in the Sankey graph, however, psychological distress remained stable throughout the whole survey period for the majority of respondents. The biggest change in psychological distress status was found at Wave 4 (November 2020), during the second wave of outbreaks when most restrictive measures had been restored. Average psychological distress decreased to 3.38 (SD = 3.78) at Wave 5 (November 2021), five months after the easing of most measures. At the time, 38% of the respondents were in psychological distress.

**Table 2** also indicates wide variation in both exposure to COVID-19 and psychosocial factors between the different waves. For example, there is greater variability in social activities (F-test=1012.0, *p* < 0.001) between the waves than in social support (F-test=39.9, *p* < 0.001).

**Table 2:** Sociodemographic characteristics of respondents and descriptive data across the five study waves (N = 4,550), COVID-and-I survey, Belgium, 2020-21.

Covariates	Wave 1 (03/2020)	Wave 2 (04/2020)	Wave 3 (06/2020)	Wave 4 (11/2020)	Wave 5 (11/2021)	F-test (p-value)^a^
	Start - first lockdown	First lockdown	Relaxation of measures	Second lockdown	Follow-up	
Psychological distress, from 0 (low) to 12 (high), (mean, std)	3.97 (3.39)	4.08 (3.75)	2.97 (3.61)	4.21 (3.95)	3.38 (3.78)	91.36 (<0.001)
Age, year (mean, std)	50.5 (14.1)	/	/	/		/
Gender, women (%) (REF=men)	72.5	/	/	/		/
Education, lower (%) (REF=higher)	11.4	/	/	/		/
Exposure to COVID-19, yes (%) (REF=no)	49.1	42.0	26.2	34.2	38.6	565.1^b^ (<0.0001)
Social Support, from 3 (low) to 14 (high) (mean, std)	9.8 (2.3)	9.8 (2.3)	10.0 (2.3)	9.4 (2.4)	9.7 (2.4)	39.9 (<0.0001)
Loneliness, from 3 (low) to 12 (high) (mean, std)	5.5 (2.4)	5.7 (2.4)	5.1 (2.1)	6.1 (2.5)	5.4 (2.3)	135.1 (<0.0001)
Social activities, from 6 (low) to 24 (high) (mean, std)	11.4 (1.9)	11.9 (2.0)	13.5 (2.2)	12.2 (1.9)	13.6 (2.4)	1012.0 (<0.0001)

a This statistical test (F-test) was used to identify any variations in sample characteristics between the different waves. ANOVA was used for continuous variables (e.g. psychological distress, social support, loneliness, and social activities), and a Chi-squared test was used for discrete variables (e.g. exposure to COVID-19).

b Chi²-test.

**Table 3** displays the outcomes of the different Bayesian linear mixed-effects models, which examine the association between multiple independent variables (including socioeconomic factors, waves, and psychosocial factors) and psychological distress, as assessed by the GHQ-12 scale. The estimated coefficients indicate the magnitude of each predictor’s effect on psychological distress. The upper section of the table describes the fixed effects, while the lower section provides the random components at the individual level.

**Table 3:** Estimates of psychological distress (GHQ-12 score) from a multivariate mixed model and fixed effects model (N = 4,550), COVID-and-I survey, Belgium, 2020-21.

	Model 1		Model 2		Model 3		Model 4	
	+ Socioeconomic factors		+ Exposure to COVID-19 virus		+ Psychosocial factors		+ Loneliness in random effect	
Covariates	Coefficients^a^	CI95%^b^	Coefficients	CI95%	Coefficients	CI95%	Coefficients	CI95%
Fixed effects								
Intercept	4.42	(3.90;4.98)	4.38	(3.82;4.90)	3.67	(3.09;4.24)	3.53	(3.34;3.71)
Gender, women, (REF=men)	0.55	(0.39;0.75)	0.56	(0.37;0.75)	0.41	(0.29;0.56)	0.41	(0.37;0.46)
Age (REF = 0–24 years old)								
25–54 years old	-0.39	(-0.91;0.13)	-0.39	(-0.88;0.14)	-0.17	(-0.55;0.27)	-0.09	(-0.22;0.05)
55+ years old	-1.62	(-2.19;-1.13)	-1.61	(-2.15;-1.11)	-1.25	(-1.65;-0.83)	-1.15	(-1.53;-0.77)
Level of education, lower (%), (REF=higher)	0.19	(-0.07;0.44)	0.18	(-0.06;0.44)	-0.36	(-0.56;-0.18)	-0.34	(-0.41;-0.28)
Waves (REF=Wave 1, March 2020)								
Wave 2, April 2020	0.12	(0.02;4.98)	0.17	(0.03;0.28)	0.15	(0.04;0.27)	0.15	(0.11;0.18)
Wave 3, June 2020	-0.99	(-1.09;-0.87)	-0.97	(-1.08;-0.86)	-0.37	(-0.48;-0.25)	-0.37	(-0.41;-0.34)
Wave 4, November 2020	0.26	(0.16;0.36)	0.30	(0.18;0.43)	-0.03	(-0.15;0.09)	-0.04	(-0.08;-0.00)
Wave 5, November 2021	-0.58	(-0.68;-0.47)	-0.53	(-0.65;-0.40)	-0.14	(-0.26;-0.01)	-0.15	(-0.19;--0.10)
Exposure to COVID-19, yes (%), (REF=no)			0.10	(-0.03;0.22)	0.22	(0.10;0.34)	0.23	(0.19;0.28)
Social support, from 3 (low) to 14 (high)					-0.18	(-0.21;-0.16)	-0.18	(-0.19;--0.17)
Loneliness, from 3 (low) to 12 (high)					0.66	(0.64;0.68)	0.65	(0.64;0.66)
Social activities, from 6 (low) to 24 (high)					-0.11	(-0.13;-0.09)	-0.11	(-0.12;-0.10)
Random effects at the individual level								
Intercept (S_0s_)	5.87	(5.57;6.17)	5.87	(5.55;6.17)	3.12	(2.94;3.30)	1.36	(1.28;1.43)
Slope: Waves (S_1s_)	1.82	(1.48;2.13)	1.79	(1.40;2.12)	1.27	(1.03;1.50)	1.16	(1.05;1.25)
Slope: Loneliness (S_2s_)							0.05	(0.05;0.06)
Residual	5.85	(5.58;6.12)	5.86	(5.60;6.20)	5.27	(5.07;5.51)	5.27	(5.04;5.51)
Model fit								
DIC^c^ (smaller is better)	111689.6		111727.5		107092.7		106953.3	

a Mean parameter of the posterior distribution.

b CI95%: credible interval 95%.

c DIC, Deviance Information Criterion.

Grey shadings means "Not applicable".

### 3.1 Fixed effects

Model 1 includes sociodemographic factors and treats different waves as fixed effects. Model 2 adds personal exposure to the COVID-19 virus, while Model 3 further includes three psychosocial variables. Models 1, 2, and 3 feature both a random intercept and a random slope (based on the waves) whereas Model 4 introduces a random slope for loneliness.

Women exhibited higher psychological distress than men (Model 1: γ=0.55, CI95% 0.39; 0.75), whereas individuals aged 55 and older experienced lower levels of psychological distress (Model 1: γ=-1.62, CI95% -2.19;-1.13). The gender difference remained relatively stable across the different models. In Models 3 and 4, however, the coefficient for women decreased from 0.55 to 0.41, which indicates that levels of psychological distress were higher among women due to their higher levels of loneliness, lower levels of social support, and fewer social activities. A similar observation was made regarding age: the protective effect of being aged 55+ increased from –1.61 to –1.25 between Model 2 and Model 3.

Greater exposure to COVID-19 was associated with a slight increase in psychological distress in Model 3 but not in Model 2 (Model 2: β=0.10, CI95% -0.03; 0.22; Model 3: β=0.22, CI95% 0.10; 0.34). Lower levels of social support, increased loneliness, and reduced social activities were all linked to higher levels of psychological distress (Models 3 and 4). Those two results, together, suggest that psychosocial variables may act as confounders in the relationship between exposure to COVID-19 and psychological distress.

The five waves were linked to fluctuations in mental health (Model 1). Notably, psychological distress was lower in Waves 3 and 5, when restrictions were lifted (β=-0.99 and -0.58, respectively), compared to Wave 1. This pattern persisted after adjusting for exposure to COVID-19 (Model 2) but weakened when psychosocial variables were included (Model 3). For example, the coefficient for Wave 3 (June 2020, associated with the lifting of most restrictive measures) increased from -0.99 (CI95% -1.09; -0.87) in Model 1 to -0.97 (CI95% -1.08; 0.86) in Model 2 and -0.37 (CI95% -0.48; -0.25) in Model 3. A similar trend was observed for Wave 4, where distress increased under restrictions but was primarily driven by psychosocial factors rather than exposure to COVID-19. Overall, these findings suggest that the variation in psychological distress between the waves was mostly influenced by social support, loneliness, and social activities, rather than by exposure to COVID-19.

### 3.2 Random effects

Interindividual variance (random intercept, S_0s_=5.87, Model 1) was larger than the intraindividual variance (random slope, S_1s_=1.82), indicating stable differences between individuals over waves. This distribution remained in Model 2. However, the inclusion of time-varying psychosocial factors in Model 3 altered the variance structure. On the one hand, interindividual variance was markedly reduced, with the random intercept decreasing from 5.87 (Model 2) to 3.12. On the other hand, intraindividual variance across waves also declined, as reflected in the reduction of the random wave slope from 1.79 (Model 2) to 1.27. Model 4, which added loneliness as a random slope, reducing the random intercept from 3.12 (Model 3) to 1.36 and the random slope from 1.27 (Model 3) to 1.16. These findings suggest that psychosocial factors largely explain interindividual differences in psychological distress but contribute less to intraindividual variation across waves.

### 3.3 Sensitivity analysis

To assess the magnitude of the loss to follow-up bias (attrition bias), our final model was replicated using all available observations, regardless of wave completion (see **Supplementary Table 1**). Overall, the fixed effects coefficients remained nearly unchanged, with minor differences: the activity coefficient decreased from –0.11 to –0.13, while the exposure to COVID-19 coefficient increased from 0.23 to 0.28. Furthermore, the random component increased from 1.39 to 1.79 for the intercept and from 1.16 to 1.36 for the wave slope.

**Figure 1:**
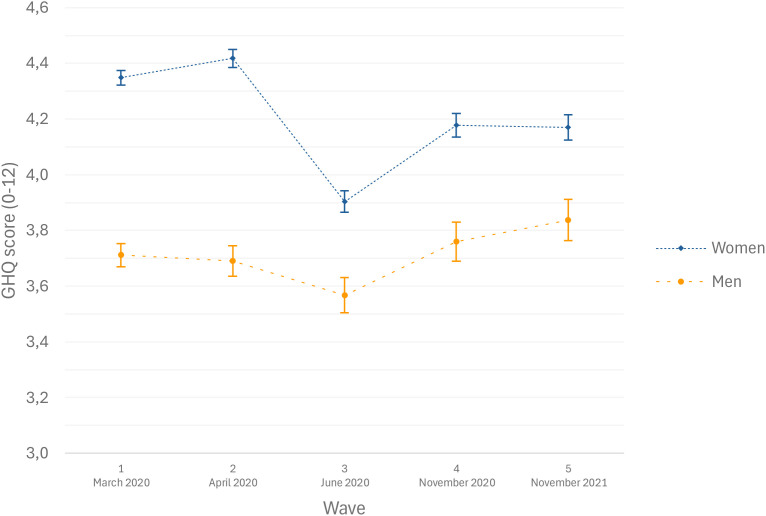
Estimates of psychological distress (GHQ-12) according to gender (N = 4,550), COVID-and-I survey, Belgium, 2020.

**Figure 2:**
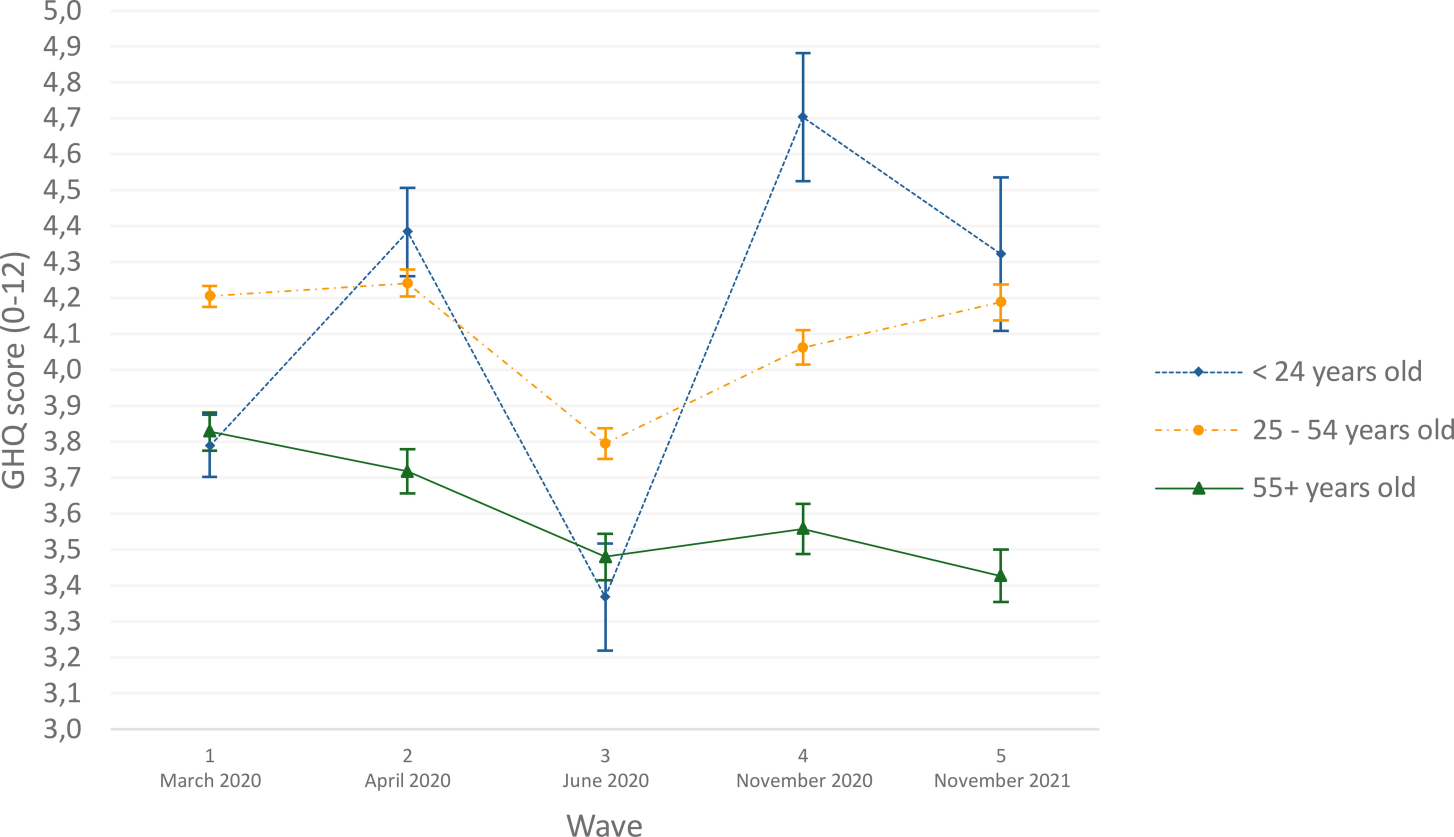
Estimates of psychological distress (GHQ-12) according to age group (N = 4,550), COVID-and-I survey, Belgium, 2020.

**Figure 1** displays psychological distress scores by gender and **Figure 2** by age, both controlling for covariates. Women consistently reported higher levels of psychological distress than men across all waves. Both genders followed a similar distress pattern, but women showed a steeper decline between April and June 2020 (contrast test F = 6.83, *p* < 0.05). Similarly, younger individuals (<24 years) demonstrated significantly higher psychological distress variations across waves than those aged 25-54, while participants aged 25–54 and over 55 maintained relatively stable levels of psychological distress. The association between loneliness and psychological distress was weaker among older respondents (aged 55 and above) compared with younger participants (interaction estimate: -0.13; 95% CI: -0.25; -0.00). In contrast, no statistically significant interaction was observed between loneliness and gender (interaction estimate: 0.02; 95% CI: -0.02; 0.07).

## 4 Discussion

In this paper, we explored how intra- and interindividual factors acted as mechanisms explaining variation in psychological distress during the pandemic in the Belgian general population during the first two years of the pandemic. Our study found that variance in psychological distress was mostly interindividual and therefore stable over time. Psychosocial factors were found to be more important contributors to psychological distress than exposure to COVID-19. Furthermore, loneliness was the main predictor of the variance between individuals in psychological distress.

### 4.1 Interindividual factors

Restrictive measures significantly impacted psychological distress, with higher levels of distress levels observed during outbreak peaks and subsequent policy restrictions, aligning with national (4, 7) and international studies (6, 12, 16, 31).

However, pandemic-related fluctuations explained only a small part of the interindividual variance in psychological distress. Including psychosocial factors in the model reduced interindividual variance estimates, indicating that loneliness, social support, and number of social activities played a larger role than COVID-19 exposure.

Consistent with previous studies (4–6, 32), women, younger people, and those with lower education levels were more vulnerable to experiencing an increase in psychological distress over time. Women reported higher psychological distress potentially attributable to multiple factors. In addition to the economic burden associated with lockdowns (8), women often assume the role of informal caregivers within families. Consequently, restrictive measures such as school and childcare facility closures likely increased caregiving and household responsibilities (33). This increased burden may have reduced women’s ability to meet work demands, heightened job insecurity, and limited employment opportunities and financial stability (34, 35). Moreover, during periods of crisis and quarantine, women have been shown to face an increased risk of exposure to domestic violence, further contributing to psychological distress (36, 37). Lastly, previous studies have highlighted that the expression of mental health disorders differs considerably between genders (38), which may partly explain observed differences in prevalence. Specifically, women tend to report higher levels of depressive feelings, whereas men more often exhibit externalizing behaviors such as excessive alcohol consumption (39). However, when considering all mental disorders combined, some authors report minimal to no gender differences across many European countries, suggesting comparable overall levels of mental health problems among men and women (38).

Younger people faced more pronounced changes in levels of distress, whereas among older people the social and occupational restrictions appeared to have a comparatively lower impact on mental health, despite their higher risk of COVID-19 related mortality (4, 7). In addition, our findings indicate that the association between loneliness and mental health was weaker among respondents aged 55 years old and older than among younger age group, suggesting greater resilience or more effective coping mechanisms in later life.

### 4.2 Intraindividual factors

This study’s originality lies in its in-depth exploration of intraindividual variance in psychological distress over time. Our longitudinal study enhances understanding of personal and temporal aspects of psychological distress. Changes in psychological distress were shaped by pandemic dynamics and policy measures, influenced by psychosocial and socioeconomic factors.

Findings align with previous research, which identified loneliness as a key determinant of psychological distress (40), and emphasized coping strategies and social factors in mental health (41). A similar French study found that increasing loneliness correlated with higher depression and anxiety (20), a link corroborated by multiple studies (42–44). Reduced social support and activity engagement also contributed to psychological distress, reinforcing the mental health impact of pandemic-induced isolation (45, 46).

### 4.3 Limitations

Despite a large overall sample size, this study is subject to selection bias arising from convenience sampling and differential attrition across waves. Women, older adults, and highly educated individuals were overrepresented compared to the Belgian general population. This could lead to both over- and underestimations of psychological distress, limiting the generalizability of prevalence estimates of psychological distress. More distressed individuals may have been more inclined to participate (4–6, 32), while at-risk groups (e.g. those with pre-existing mental conditions) may have been underrepresented. As a result, the absolute levels of psychological distress and their evolution over time should be interpreted with caution.

Due to the concern of a selection bias related to uneven selection in the baseline survey, we compared the relationship between psychological distress and social support in a 2018 random sample with our current baseline data (4). The association remained nearly identical between the two data collections. We also conducted a sensitivity analysis using all available observations rather than restricting analyses to complete cases. The results showed that the direction and relative magnitude of the fixed effects remained largely unchanged, with only minor differences in coefficient size, suggesting that the estimated associations between psychosocial factors, COVID-19 exposure, and psychological distress were robust to attrition. In addition, we used mixed-effects models with random intercepts and random slopes for wave and loneliness to account for individual differences in baseline levels of psychological distress and in how loneliness is associated with distress over time. By explicitly modelling this baseline and longitudinal individual-level heterogeneity, this approach limits the undue influence of individual-specific variation on population-level estimates. This enhances the robustness of our findings and reduces the risk of bias in their interpretation.

## 5 Conclusions

Changes in psychological distress in the general population are influenced by pandemic dynamics, policy measures, and individual characteristics. Consistent with our findings, women, young people, and those with lower education levels are particularly vulnerable, while loneliness, reduced social support, and fewer activities appear to exacerbate psychological distress.

Although many containment policies have taken a sectoral approach, these measures may have unintended mental health consequences. Despite a non-probabilistic sample, findings from our study suggest that interventions could potentially be tailored to sociodemographic factors to support mental health. Shorter lockdowns and social distancing periods, alongside mental health initiatives like virtual peer support groups and awareness campaigns, may help mitigate distress (47). Comparative research across countries is needed to more robustly evaluate the impact of different policy measures and their stringency.

In conclusion, addressing loneliness is crucial for preventing common mental health problems post-pandemic (20). Interventions that consider individual vulnerabilities rather than focusing solely on pandemic dynamics, may help inform more supportive strategies during and beyond crisis, while recognizing that these recommendations are based on observational data and non-random sampling.

## Data Availability

The raw data supporting the conclusions of this article will be made available by the authors, without undue reservation.
