# Supplementary material for: Dynamics of psychological distress: understanding the impact of intraindividual and interindividual factors in the Belgian population during the COVID-19 pandemic—a multilevel prospective cohort study
Source: Front Psychiatry. 2026 Mar 6;17:1716253. doi: 10.3389/fpsyt.2026.1716253 (PMC13002600; doi:10.3389/fpsyt.2026.1716253)

Supplementary material

Supplementary Table 1 Effect of fixed and random effects on psychological distress (N= 56,308 in 22750 unique individuals)

|  | Model 4 | |
| --- | --- | --- |
|  | + Loneliness in random effect | |
| Covariates | Coefficientsa | CIb95% |
| Fixed effects |  |  |
| Intercept | 3.72 | (3.46;3.98) |
| Gender, women, (REF=men) | 0.60 | (0.52;0.67) |
| Age (REF=0-24 years old) |  |  |
| 25-54 years old | -0.22 | (-0.35;-0.08) |
| 55+ years old | -1.24 | (-1.39;-1.10) |
| Level of education, lower (%), (REF=higher) | -0.21 | (-0.31;-0.11) |
| Waves (REF=Wave 1, March 2020) |  |  |
| Wave 2, April 2020 | 0.14 | (0.08;0.21) |
| Wave 3, June 2020 | -0.43 | (-0.51;-0.36) |
| Wave 4, November 2020 | -0.04 | (-0.12;0.04) |
| Wave 5, November 2021 | -0.06 | (-0.15;0.03) |
| Exposure to COVID-19, yes (%), (REF=no) | 0.28 | (0.22;0.35) |
| Social support, from 3 (low) to 14 (high) | -0.17 | (-0.19;-0.16) |
| Loneliness, from 3 (low) to 12 (high) | 0.66 | (0.64;0.67) |
| Social activities, from 6 (low) to 24 (high) | -0.13 | (-0.14;-0.11) |
| Random effects at the individual level |  |  |
| Intercept (S_0s_) | 1.79 | (1.65;1.93) |
| Slope: Waves (S_1s_) | 1.36 | (1.22;1.49) |
| Slope: Loneliness (S_2s_) | 0.05 | (0.04;0.05) |
| Residual | 5.32 | (5.19;5.44) |

^a^ Mean parameter of the posterior distribution
^b^ CI95%: credible interval 95%

Supplementary Table 2 Interaction effects of loneliness with age and gender on psychological distress (GHQ-12 score) from a multivariate mixed model (N=4,550), COVID-and-I survey, Belgium, 2020-21

|  | Model 3 | | Model 3B | |
| --- | --- | --- | --- | --- |
|  | + Psychosocial  factors | | + Psychosocial factors  + Interactions loneliness*gender/age | |
| Covariates | Coefficients^a^ | CI95%^b^ | Coefficients | CI95% |
| Fixed effects |  |  |  |  |
| Intercept | 3.67 | (3.09;4.24) | 3.12 | (2.18;4.08) |
| Gender, women, (REF=men) | 0.41 | (0.29;0.56) | 0.30 | (0.11;0.35) |
| Age (REF=0-24 years old) |  |  |  |  |
| 25-54 years old | -0.17 | (-0.55;0.27) | 0.40 | (-0.47;1.25) |
| 55+ years old | -1.25 | (-1.65;-0.83) | -0.48 | (-1.34;0.38) |
| Level of education, lower (%), (REF=higher) | -0.36 | (-0.56;-0.18) | -0.37 | (-0.56;-0.17) |
| Waves (REF=Wave 1, March 2020) |  |  |  |  |
| Wave 2, April 2020 | 0.15 | (0.04;0.27) | 0.15 | (0.04;0.27) |
| Wave 3, June 2020 | -0.37 | (-0.48;-0.25) | -0.36 | (-0.48;-0.25) |
| Wave 4, November 2020 | -0.03 | (-0.15;0.09) | -0.03 | (-0.15;0.09) |
| Wave 5, November 2021 | -0.14 | (-0.26;-0.01) | -0.14 | (-0.26;-0.01) |
| Exposure to COVID-19, yes (%), (REF=no) | 0.22 | (0.10;0.34) | 0.23 | (0.11;0.35) |
| Social support, from 3 (low) to 14 (high) | -0.18 | (-0.21;-0.16) | -0.18 | (-0.21;-0.16) |
| Loneliness, from 3 (low) to 12 (high) | 0.66 | (0.64;0.68) | 0.74 | (0.62;0.87) |
| Social activities, from 6 (low) to 24 (high) | -0.11 | (-0.13;-0.09) | -0.11 | (-0.13;-0.09) |
| Loneliness*Women (REF=men) |  |  | 0.02 | (-0.02;0.07) |
| Loneliness*25-54 years old (REF=0-24 years old) |  |  | -0.09 | (-0.21;0.04) |
| Loneliness*55+ years old (REF=0-24 years old) |  |  | -0.13 | (-0.25;-0.00) |
| Random effects at the individual level |  |  |  |  |
| Intercept (S_0s_) | 3.12 | (2.94;3.30) | 3.12 | (2.18;4.08) |
| Slope: Waves (S_1s_) | 1.27 | (1.03;1.50) | 1.23 | (0.96;1.57) |
| Slope: Loneliness (S_2s_) |  |  |  |  |
| Residual | 5.27 | (5.07;5.51) | 5.30 | (5.02;5.55) |
| Model fit |  |  |  |  |
| DIC^c^ (smaller is better) | 107092.7 |  | 107118.2 |  |

^a^ Mean parameter of the posterior distribution
^b^ CI95%: credible interval 95%
^c^ Deviance Information Criterion

Supplementary Figure 1 Sankey graph of the variation in psychological distress (GHQ-12 score ≥ 4) across the five study waves (N=4,550), COVID-and-I survey, Belgium, 2020-21. Alt text: A Sankey graph illustrating variations in psychological distress (GHQ-12 score >=4) across five study waves in the COVID-and-I survey. Light blue represents participants with GHQ-12 scores >=4 (indicating distress), while dark blue represents those with score <4. Flows between waves show transitions in distress levels over time within the study population.


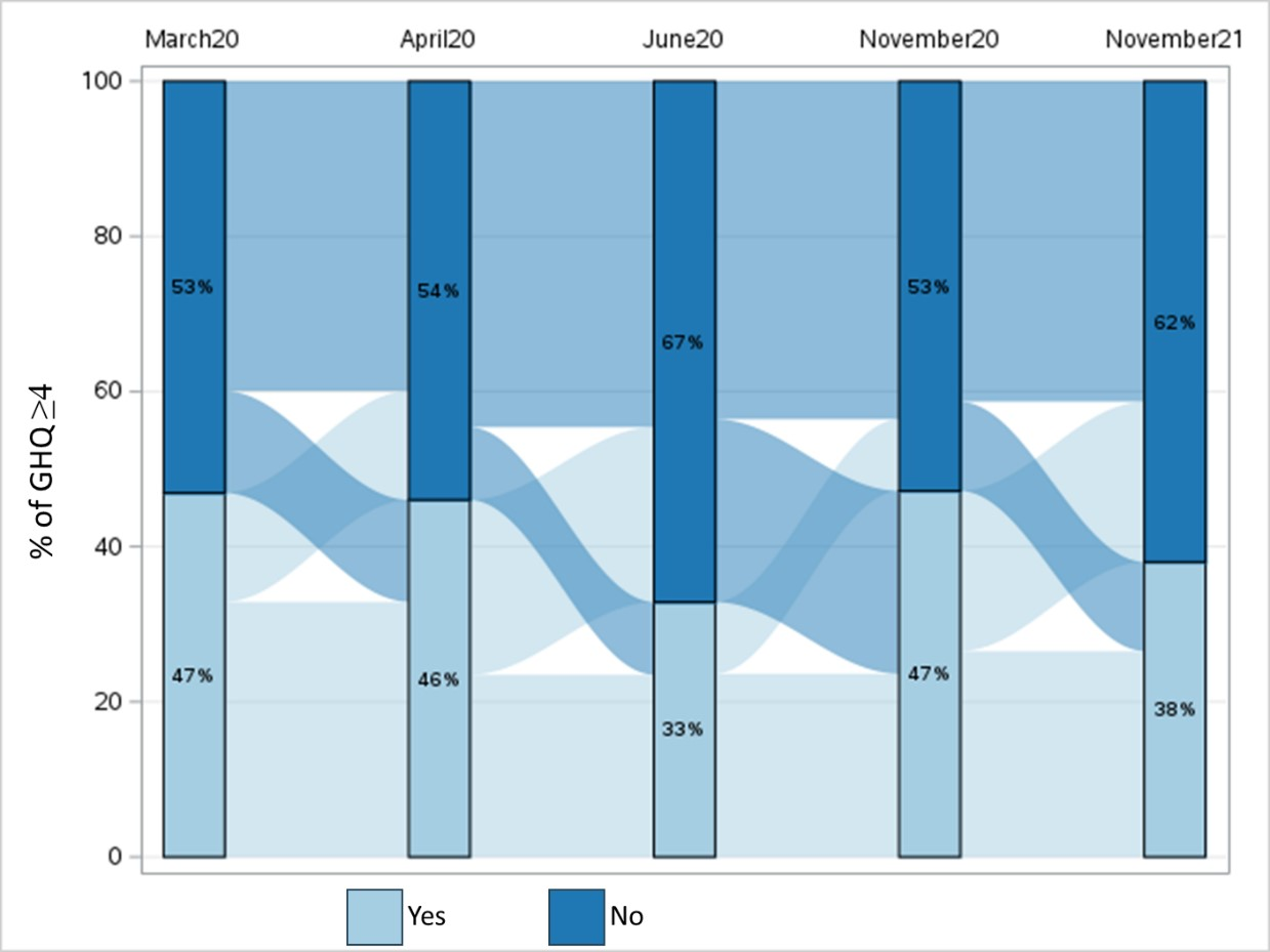

Supplement: Supplementary file 1 [file Supplementaryfile1.docx]
